# Quantitative Expression Analysis of APP Pathway and Tau Phosphorylation-Related Genes in the ICV STZ-Induced Non-Human Primate Model of Sporadic Alzheimer’s Disease

**DOI:** 10.3390/ijms16022386

**Published:** 2015-01-22

**Authors:** Sang-Je Park, Young-Hyun Kim, Gyu-Hwi Nam, Se-Hee Choe, Sang-Rae Lee, Sun-Uk Kim, Ji-Su Kim, Bo-Woong Sim, Bong-Seok Song, Kang-Jin Jeong, Youngjeon Lee, Young Il Park, Kyoung-Min Lee, Jae-Won Huh, Kyu-Tae Chang

**Affiliations:** 1 National Primate Research Center, Korea Research Institute of Bioscience and Biotechnology, Chungbuk 363-883, Korea; E-Mails: parksj@kribb.re.kr (S.-J.P.); kyh@kribb.re.kr (Y.-H.K.); nkh14@kribb.re.kr (G.-H.N.); csh91@kribb.re.kr (S.-H.C.); srlee@kribb.re.kr (S.-R.L.); sunuk@kribb.re.kr (S.-U.K.); kimjs@kribb.re.kr (J.-S.K.); embryont@kribb.re.kr (B.-W.S.); sbs6401@kribb.re.kr (B.-S.S.); nemo9426@kribb.re.kr (K.-J.J.); neurosci@kribb.re.kr (Y.L.); 2 University of Science & Technology, National Primate Research Center, Korea Research Institute of Bioscience and Biotechnology, Chungbuk 363-883, Korea; 3 Graduate School Department of Digital Media, Ewha Womans University, Seoul 120-750, Korea; E-Mail: yipark@ewha.ac.kr; 4 Department of Neurology, Seoul National University Hospital, Seoul 110-744, Korea; E-Mail: kminlee@snu.ac.kr

**Keywords:** Alzheimer’s disease, streptosozocin, cynomolgus monkey, qPCR, APP, tau

## Abstract

The accumulation and aggregation of misfolded proteins in the brain, such as amyloid-β (Aβ) and hyperphosphorylated tau, is a neuropathological hallmark of Alzheimer’s disease (AD). Previously, we developed and validated a novel non-human primate model for sporadic AD (sAD) research using intracerebroventricular administration of streptozotocin (icv STZ). To date, no characterization of AD-related genes in different brain regions has been performed. Therefore, in the current study, the expression of seven amyloid precursor protein (APP) pathway-related and five tau phosphorylation-related genes was investigated by quantitative real-time PCR experiments, using two matched-pair brain samples from control and icv STZ-treated cynomolgus monkeys. The genes showed similar expression patterns within the control and icv STZ-treated groups; however, marked differences in gene expression patterns were observed between the control and icv STZ-treated groups. Remarkably, other than β-secretase (*BACE1*) and cyclin-dependent kinase 5 (*CDK5*), all the genes tested showed similar expression patterns in AD models compared to controls, with increased levels in the precuneus and occipital cortex. However, significant changes in gene expression patterns were not detected in the frontal cortex, hippocampus, or posterior cingulate. Based on these results, we conclude that APP may be cleaved via the general metabolic mechanisms of increased α- and γ-secretase levels, and that hyperphosphorylation of tau could be mediated by elevated levels of tau protein kinase, specifically in the precuneus and occipital cortex.

## 1. Introduction

Sporadic Alzheimer’s disease (sAD) is the most common neurodegenerative disease in the human population. More than 35 million people have AD worldwide, which is clinically characterized by the deterioration of memory and cognitive function [1]. The majority of AD cases are sAD (≥95%), and result from neuronal loss, accumulation of senile plaques consisting of amyloid-β peptide (Aβ), aggregation of neurofibrillary tangles consisting of hyperphosphorylated tau protein, and brain atrophy [2]. Approximately 5% of AD cases are caused by missense mutations of three genes: presenilin 1 (*PSEN1*), *PSEN2*, and amyloid precursor protein (*APP*), leading to the accumulation of Aβ and disease onset before the age of 65 [3,4]; this type of AD is known as early-onset familial AD (fAD). In previous studies, many molecular alterations were also identified in sAD, including activation of pro-death genes and signaling pathways, mitochondrial dysfunction, impairment of energy metabolism, synaptic dysfunction, and oxidative and inflammatory damage [1,5]. These changes have been correlated with the accumulation of misfolded Aβ and hyperphosphorylated tau proteins in the aging brain.

To investigate the molecular and pathological mechanisms of sAD, intracerebroventricular injection of streptozotocin (icv-STZ) rodent animal models were established. These animals showed similar pathological features to sAD, such as neuronal loss, impairment of spatial learning, accumulation of Aβ, increased hyperphosphorylation of tau proteins, and a chronic decrease of cerebral glucose uptake and production [6,7]. Importantly, abnormalities in brain glucose metabolism are a major feature of the early stages of sAD [8]. Therefore, the icv-STZ rodent models are useful animal models for the investigation of sAD. Expression levels of both mRNA transcripts and proteins of insulin signaling pathway-related genes, AD-related genes (such as *APP*, *tau*, and β-site APP cleaving enzyme 1 (*BACE1*)), and other genes have been previously investigated in the brains of icv STZ-treated animals [6,9]. However, it can be difficult to investigate the spatial distribution and regional differences in pathogenetic vulnerability in rodent models, as the small rodent brain does not allow detailed spatial mapping. For instance, to investigate the expression of mRNA and proteins of target genes, only one or three brain samples (corresponding to different regions) were used in the studies using STZ rodent models [3,6,9,10]. Moreover, rodents do not spontaneously form accumulations of Aβ in the brain, and the sequence of the rodent Aβ peptide is not identical to that of the human [11]. Thus, although the usefulness of the STZ rodent model is undisputed, additional relevant information regarding the pathological mechanisms of sAD may be obtained from other appropriate animal models. To better understand spatial- and regional-specific differences in the pathological and molecular biological features of sAD, we established a primate model of sAD by icv injection of STZ in the cynomolgus monkey (*Macaca fascicularis*) [12]. The cynomolgus monkey has several advantages (e.g., genetic, morphological, physiological, and behavioral similarities to humans) for the investigation of neurodegenerative diseases. Furthermore, this animal has been shown to demonstrate Aβ deposition, tau accumulation, and neurofibrillary tangle formation in the brain in an age-dependent and region-specific manner, similar to that in humans [13,14]. Interestingly, icv STZ-treated cynomolgus monkeys show a region-specific decrease in glucose metabolism in the precuneus, posterior cingulate, and medial temporal cortices by fluorodeoxyglucose-positron emission tomography (FDG-PET) imaging, after icv-STZ injection at 6 and 12 weeks, similar to the early stages of sAD in patients [8]. This study, however, did not include molecular characterization of AD-related genes. Therefore, to investigate altered gene expression in detail, samples from the brains of icv STZ-treated non-human primates are needed.

In this study, we performed quantitative expression analysis of APP pathway-related genes including α-secretases (Assignment of a disintegrin and metallopeptidase domain 10 (*ADAM10*) and *ADAM17*), *BACE1*, and γ-secretases (presenilin2 (*PSEN2*), nicastin (*NCSTN*), anterior pharynx defective 1 homolog A (*APH1A*), presenilin enhancer 2 homolog (*PSENEN*)) and tau phosphorylation-related genes such as cyclin-dependent kinase 5 (*CDK5*), cyclin-dependent kinase 5 regulatory subunit 1 (*p35*) (*CDK5R1*), calpain 1, (mu/I) large subunit (*CAPN1*), v-akt murine thymoma viral oncogene homolog 1 (*AKT1*), and glycogen synthase kinase 3 beta (*GSK3β*) using RT-qPCR experiments.

## 2. Results

### 2.1. Relative Expression Analysis of Amyloid Precursor Protein (APP) Pathway-Related Genes

**Figure 1:**
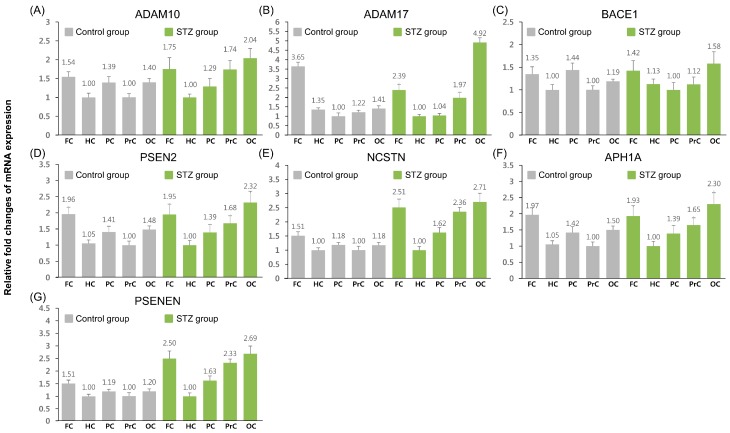
Quantitative expression analysis of amyloid precursor protein (APP) pathway-related genes in control and intracerebroventricular administration of streptozotocin (icv-STZ) groups. Quantification data for all genes were normalized using appropriate reference genes (control group: glyceraldehyde-3-phosphate dehydrogenase (*GAPDH*) and ribosomal protein S (*RPS19*) and icv-STZ group: β-actin (*ACTB*) and *GAPDH*) and relative fold changes of expression level was calculated by lowest expressed tissue in each gene. Data are expressed as means ± SD. FC, frontal cortex; HC, hippocampus; PC, posterior cingulate; PrC, precuneus; OC, occipital cortex. (**A**) Assignment of a disintegrin and metallopeptidase domain 10 (*ADAM10*); (**B**) *ADAM17*; (**C**) β-site APP cleaving enzyme 1 (*BACE1*); (**D**) presenilin2 (*PSEN2*); (**E**) nicastin (*NCSTN*); (**F**) anterior pharynx defective 1 homolog A (*APH1A*); (**G**) presenilin enhancer 2 homolog (*PSENEN*).

Relative mRNA expression levels of seven APP pathway-related genes were measured by RT-qPCR. These genes are part of the enzymatic machinery of α-, β-, and γ-secretases, which are involved in the cleavage of APP; *ADAM10* and *ADAM17* encode α-secretases, *BACE1* encodes a β-secretase, and *PSEN2*, *NCSTN*, *APH1A*, and *PSENEN* belong to the γ-secretase family. First, we investigated the mRNA expression pattern of these genes in the control and icv STZ-treated groups (Figure 1). In the control group, all genes, with the exception of *ADAM17*, showed similar expression patterns and were more abundantly expressed in the frontal, posterior cingulate, and occipital cortices than in the hippocampus and the precuneus. Significantly increased expression of *ADAM17* was observed in the frontal cortex compared to the other tissues examined.

In the icv STZ-treated group, a different expression pattern was observed when compared to the control group, and within the group, all genes demonstrated similar expression patterns. Expression levels were significantly increased in the frontal cortex, precuneus, and occipital cortex compared to the hippocampus and posterior cingulate.

**Figure 2:**
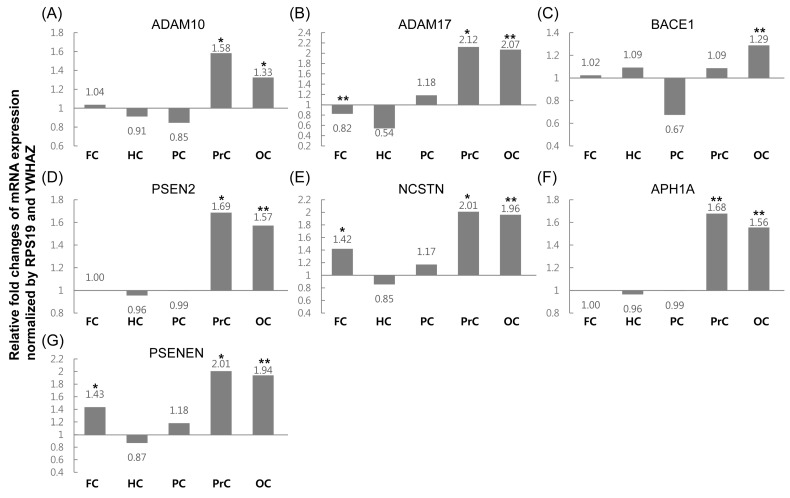
mRNA levels of amyloid precursor protein (APP) pathway-related genes in the five selected brain areas of icv-STZ monkeys relative to levels in normal monkeys were assessed by quantitative real-time PCR (FC, frontal cortex; HC, hippocampus; PC, posterior cingulate; PrC, precuneus; OC, occipital cortex). (**A**–**G**) relative expression of *ADAM10*, *ADAM17*, *BACE1*, *PSEN2*, *NCSTN*, *APH1A*, and *PSENEN*, respectively. Two cynomolgus monkeys per group were analyzed in triplicate, and the values were normalized to the geometric mean of two optimal reference genes, tyrosine 3-monooxygenase/tryptophan 5-monooxygenase activation protein ζ polypeptide (*YWHAZ*) and *RPS19*, using the Relative Expression Software Tool (REST) 2009. The asterisks indicate a statistical difference (* *p* < 0.05 and ** *p* < 0.001) by randomization tests of REST 2009. Comparative expression levels of each gene in the different brain areas and total data was derived from combination of quantification cycle (Cq) values in five brain regions.

The relative fold change of mRNA expression levels of the seven genes was compared between samples from icv STZ-treated and control animals (Figure 2). Almost all genes demonstrated significantly increased expression levels in the precuneus and occipital cortex (approximately 1.6–2.1-fold) compared to the control. In contrast, *BACE1* was only increased approximately 1.3-fold in the occipital cortex. In the frontal cortex, expression levels of *NCSTN* and *PSENEN* were increased (approximately 1.4-fold) and *ADAM17* was decreased (0.82-fold). No remarkable differences in the expression levels of any gene were observed in the hippocampus and the posterior cingulate.

### 2.2. Relative Expression Analysis of Tau Phosphorylation-Related Genes

**Figure 3:**
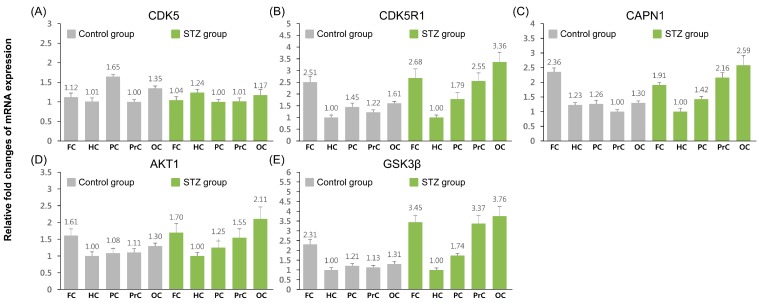
Quantitative expression analysis of tau phosphorylation-related genes in control and icv-STZ groups. Quantification data for all genes were normalized using appropriate reference genes (control group: *GAPDH* and *RPS19* and icv-STZ group: *ACTB* and *GAPDH*) and relative fold changes of expression level was calculated by lowest expressed tissue in each gene. Data are expressed as means ± SD. FC, frontal cortex; HC, hippocampus; PC, posterior cingulate; PrC, precuneus; OC, occipital cortex. (**A**) cyclin-dependent kinase 5 (*CDK5*); (**B**) cyclin-dependent kinase 5 regulatory subunit 1 (*p35*) (*CDK5R1*); (**C**) calpain 1, (mu/I) large subunit (*CAPN1*); (**D**) v-akt murine thymoma viral oncogene homolog 1 (*AKT1*); (**E**) glycogen synthase kinase 3 beta (*GSK3β*).

Relative mRNA expression levels of five tau phosphorylation-related genes were also measured in the control and icv STZ-treated groups (Figure 3). Other than *CDK5*, the genes showed similar expression patterns across the control group, with high expression levels in the frontal cortex compared to other regions. Expression levels in other regions showed only minor differences, including higher levels of *CDK5R1* in the posterior cingulate and occipital cortex, and of *CDK5* and *GSK3β* in the occipital cortex alone. Elevated expression levels of *CDK5* were observed in the posterior cingulate and hippocampus.

In the icv STZ-treated group, there were similar patterns of gene expression across regions, with the exception of *CDK5*. High expression levels of all other genes were observed in the frontal cortex, precuneus, and occipital cortex, and low levels of transcription were observed in the hippocampus; *CDK5* was expressed at similar levels in all regions.

**Figure 4:**
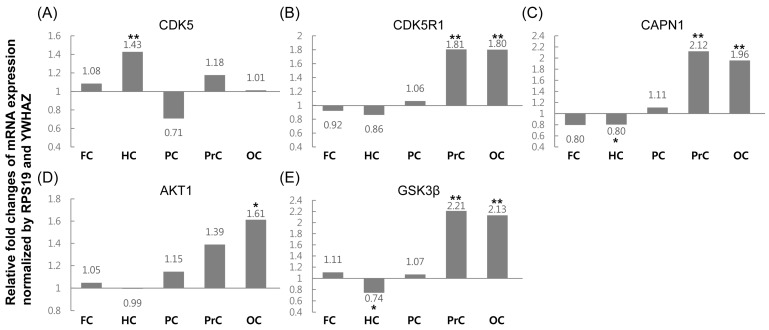
mRNA levels of tau phosphorylation-related genes in the five selected brain areas of icv-STZ monkeys relative to levels in normal monkeys were assessed by quantitative real-time PCR (FC, frontal cortex; HC, hippocampus; PC, posterior cingulate; PrC, precuneus; OC, occipital cortex). (**A**–**E**) relative expression of *CDK5*, *CDK5R1*, *CAPN1*, *AKT1* and *GSK3β*, respectively. Two cynomolgus monkeys per group were analyzed in triplicate, and the values were normalized to the geometric mean of two optimal reference genes, YWHAZ and RPS19, using the Relative Expression Software Tool (REST) 2009. The asterisks indicate a statistical difference (* *p* < 0.05 and ** *p* < 0.001) by randomization tests of REST 2009. Comparative expression levels of each gene in the different brain areas and total data was derived from combination of Cq values in five brain regions.

Relative fold changes of mRNA expression levels of the five genes were compared between the icv STZ-treated and control groups (Figure 4). The expression levels of *CDK5R1*, *CAPN1*, and *GSK3β* were similar; levels in the precuneus and occipital cortex were significantly increased (approximately 1.8–2.2-fold) in the icv STZ-treated group compared to that in the controls, and the expression levels of *CAPN1* and *GSK3β* in the hippocampus were decreased (approximately 1.2–1.4-fold). In the case of *AKT1*, although expression patterns were similar to those of *CDK5R1*, *CAPN1*, and *GSK3β*, levels in the occipital cortex were increased (about 1.6-fold) in the icv STZ-treated group compared to the controls. By contrast, different expression patterns were only observed for *CDK*; its expression level was only increased by 1.43-fold in the hippocampus.

## 3. Discussion

Gene expression analysis is a powerful experimental approach and can contribute to the basic understanding of genetic mechanisms underlying various environmental responses. The RT-qPCR technique is a very powerful and sensitive method to detect and quantify the transcription of target genes due to its specificity, accuracy, and broad range of application [15,16,17]. Therefore, we used RT-qPCR to analyze the expression levels of AD-related genes, including those in the APP pathway and tau phosphorylation-related genes, in brain samples (five regions) from animals treated with icv-STZ to induce sAD, and we compared them to levels measured in the control group.

First, we investigated the expression pattern of 12 APP pathway-related and tau phosphorylation-related genes in the control and icv STZ-treated groups (Figure 1 and Figure 3). In the control group, the majority of genes showed high levels of expression in the frontal cortex, except for *BACE1* and *CDK5*. Moreover, expression patterns were similar among the genes of each pathway, except for *ADAM17* and *CDK5*. These results indicate that genes of the APP pathway-related group and tau phosphorylation-related group seem to be maintained at similar levels under normal conditions. In the icv STZ-treated group, all the genes showed high levels of transcription in the frontal and occipital cortices, and their expression patterns were similar to others of the same pathway, except for *CDK5*. Moreover, major alterations of gene expression patterns were observed between the control and icv STZ-treated groups. These results indicate that all the genes included in the study are likely to be regulated similarly across regions, and that their expression levels could be affected by injecting icv-STZ. In the case of *BACE1* and *CDK5*, no remarkable changes in expression level were observed in any region, indicating that the expression of these genes may not be affected by icv-STZ treatment.

Finally, we measured the fold change in mRNA expression of APP pathway-related and tau phosphorylation-related genes in the icv STZ-treated group compared with the control group (Figure 2 and Figure 4). All of the APP pathway-related genes demonstrated high expression levels in the precuneus and occipital cortex, except for *BACE1* (Table 1). No statistically significant difference in *BACE1* expression level was detected in any region, except for the occipital cortex. These results agree with those reported from studies using STZ-injected 5X Familial Alzheimer’s Disease (5XFAD) mice [18]. In our previous study, increased levels of *APP* expression were observed in the precuneus (approximately 2.2-fold) and occipital cortex (approximately 1.4-fold) in the icv STZ-treated group compared to controls, and the expression levels in other regions were also slightly changed (Table 1) [19]. These phenomena could be explained if increased APP protein levels are cleaved by general metabolic processes, consisting of increased α- and γ-secretase levels, but not β-secretase levels, in the precuneus and occipital cortex. This hypothesis is based on the observation that α- and γ-secretase-related genes showed similar expression levels and patterns to those of *APP*, whereas the transcription of β-secretase was similar to that of the controls in these regions. The biological function of APP is synaptic formation and repair, and its expression levels are upregulated during neuronal differentiation and after neural injury [20,21]; however, over-expression of APP may increase the risk of AD through the impairment of long-term potentiation, which increases susceptibility to ischemic brain damage in rodents [22,23]. Therefore, further functional studies are needed to understand the effects of increased expression levels of APP in the precuneus and occipital cortex.

**Table 1:** Relative fold change of mRNA expression of all genes in five tissues of intracerebroventricular administration of streptozotocin (icv STZ) models compared to normal controls.

Gene	Frontal Cortex	Hippocampus	Posterior Cingulate	Precuneus	Occipital Cortex
*APP*	1.14	0.94	↑ 1.23 *	↑ 2.17 *	↑ 1.44 **
*ADAM10*	1.04	0.91	0.85	↑ 1.58 *	↑ 1.33 *
*ADAM17*	↓ 0.82 **	0.54	1.18	↑ 2.12 *	↑ 2.07 **
*BACE1*	1.02	1.09	0.67	1.09	↑ 1.29 *
*PSEN2*	1.00	0.96	0.99	↑ 1.69 *	↑ 1.57 **
*NCSTN*	↑ 1.42 *	0.85	1.17	↑ 2.01 *	↑ 1.96 **
*APH1A*	1.00	0.96	0.99	↑ 1.68 **	↑ 1.56 **
*PSENEN*	↑ 1.43 *	0.87	1.18	↑ 2.01 *	↑ 1.94 **
*TAU*	1.14	1.10	0.96	1.18	1.21
*CDK5*	1.08	↑ 1.43 **	0.71	1.18	1.01
*CDK5R1*	0.92	0.86	1.06	↑ 1.81 **	↑ 1.80 **
*CAPN1*	0.80	0.80 *	1.11	↑ 2.12 **	↑ 1.96 **
*AKT1*	1.05	0.99	1.15	1.39	↑ 1.61 **
*GSK3β*	1.11	↓ 0.74 *	1.07	↑ 2.21 **	↑ 2.13 **

Bold values are statistically significant (* *p* < 0.05 and ** *p* < 0.01). Expression level of *APP* and *TAU* genes derived from our previous report [19]. ↓, decrease fold change; ↑, increase fold change.

Hyperphosphorylated tau is a major risk factor for sAD, due to its insolubility and tendency to aggregate with other hyperphosphorylated tau molecules. These abnormally aggregated tau molecules are cytotoxic and are associated with the impairment of cognition [1]. The genes *CDK5*, *CDK5R1*, *CAPN1*, *AKT1*, and *GSK3β* are involved in the regulation of tau phosphorylation. CDK5 is able to induce an increase in tau phosphorylation and neurodegeneration, although the monomeric form of CDK5 is enzymatically inactive [24]. CDK5R1 (p35) is a neuron-specific activator of CDK5 and may be proteolytically cleaved by CAPN1 (to form the more stable form of CDK5R1 (p25)) [25]. The formation of heterodimers including CDK5 and the stable form of CDK5R1 (p25) may cause the phosphorylation of tau proteins. GSK3β is another major kinase involved in tau hyperphosphorylation [26], and AKT1 is an upstream negative regulator for phosphorylation of the *N*-terminal serine of GSK3β [27]. Our results indicate similar expression patterns for *CDK5R1*, *CAPN1*, and *GSK3β*, with higher levels in the precuneus and occipital cortex than other tissues (Figure 4 and Table 1). *AKT1* expression patterns also showed a pattern similar to these three genes; however, expression levels were only slightly increased in the precuneus and occipital cortex. Therefore, AKT1 could not effectively regulate GSK3β phosphorylation. The expression pattern of *CDK5* was different from that of the other genes, and levels did not differ between regions, except for the hippocampus and posterior cingulate. In our previous study, we demonstrated that the expression levels of the *tau* gene were only slightly altered between the control and icv STZ-treated groups (Table 1) [19]. The expression levels of tau protein showed no change after injection of icv-STZ; hyperphosphorylated tau could accumulate due to the actions of activated kinase proteins such as the CDK5-CDK5R1 (p25) complex and GSK3β in the precuneus and occipital cortex. Further functional studies are needed to demonstrate this effect, such as measurement of hyperphosphorylated tau levels in the precuneus and occipital cortex. In addition, as many types of protein kinases have been reported to be involved with tau phosphorylation [28], quantitative expression analysis of these genes in the icv-STZ monkey model will be required.

To understand the pathological mechanisms of sAD, previous studies have used non-transgenic rodent models involving icv injection of STZ and subsequent quantitative analysis of AD-related genes in brain regions such as the hippocampus, temporal lobe, and cerebral cortex [6,9,29,30]. No remarkable expression level changes of AD-related genes, including *APP*, *ADAM9*, *BACE1*, *APH1A*, *PSEN2*, *tau*, *CDK5*, *CAPN1*, and *GSK3β*, in the hippocampus of icv STZ-treated mice were found [30]. On the contrary, a significantly decreased *AKT1* expression level was detected in this region [6]. Another study showed that an increased expression level of *BACE1* was detected in the cerebral cortex of STZ-treated rats (1.5-fold increase) [9]. In the present study, quantitative analysis showed different results than those obtained from the rodent model. The expression level of APP pathway-related genes, *CDK5R1*, and *AKT1* was not changed in the hippocampus, whereas statistically significant changes of expression level were detected in *CDK5* (1.43-fold increase), *CAPN1* (1.25-fold decrease), and *GSK3β* (1.35-fold decrease). The difference found among the studies could be from the use of different animal models or differing effects of injecting STZ between species. However, we think that these differing results between monkey and rodent models may affect our understanding of the molecular and pathological mechanisms underlying sAD. Therefore, to avoid confusion from analyzing the results from two distinct animal models, we believe that the icv STZ-treated cynomolgus monkey, which has many advantages such as genetic, morphological, physiological, and behavioral similarities to humans compared to the rodent model, should be used as the model to investigate sAD.

## 4. Experimental Section

### 4.1. Experimental Animals and Sampling

Four healthy 3-year-old, 3–4 kg female cynomolgus monkeys were used, originating from Vietnam, and imported from China with the Convention on International Trade in Endangered Species of Wild Fauna and Flora (CITES) permit. All animals were provided by the National Primate Research Center (NPRC) of Korea. In our experiments, specific pathogen-free (SPF) animals were used. All animals underwent a complete physical, viral, bacterial, and parasite examination. On physical examination, SPF animals were examined using various criteria, including coat condition, appearance, weight, sex, and date of birth. An enzyme immunoassay was performed to detect viruses such as simian herpes B virus (BV); simian T-cell lymphotropic/leukemia virus (STLV)-1 and -2; simian immunodeficiency virus (SIV); simian retrovirus (SRV)-1, -2, and -5; and simian varicella virus (SVV). In addition, tests were performed to detect *Mycobacterium tuberculosis* (TB), *Shigella* spp., *Salmonella* spp., and *Yersinia* spp. For the TB skin test, all animals were tested by an intradermal injection in the eyelid, and the remaining bacterial examination items were checked by fecal culture tests. In our SPF animals, all items in the above tests were negative. The monkeys were kept indoors in individual cages and fed commercial monkey chow2 (Harlan) supplemented daily with various fruits, and supplied water *ad libitum*. Environmental conditions were controlled to provide a temperature of 24 ± 2 °C, a relative humidity of 50% ± 5%, 100% fresh air at a rate of ≥12 room changes per hour, and a 12:12 h light:dark cycle. The monkey was given access to environmental enrichment such as approved toys, perches, or music to promote psychological well-being. Their health was monitored by the attending veterinarian consistent with the recommendations of the Weatherall Report.

All experimental animals were derived from our previous study [12]. The four cynomolgus monkeys were divided into two groups; the icv-STZ group (*n* = 2) and the control group (*n* = 2). In the icv-STZ group, STZ was injected into the cerebrospinal fluid (CSF) via the cerebellomedullary cistern (CM) using a 25 gauge spinal needle on days 1, 7, and 14. The monkeys were treated with 2 mg/kg STZ dissolved in 0.3 mL of normal saline. The control monkeys were injected with the same volume of normal saline. Animals were sacrificed at 20 weeks after the STZ or saline treatments, following deep anesthesia using ketamine (20 mg/kg) by intramuscular injection and perfusion with diethylpyrocarbonate (DEPC)-treated cold phosphate buffered saline (PBS) *via* the common carotid artery with RNase inhibitors, to inhibit blood contamination and promote recovery of intact RNA molecules from the tissue samples.

### 4.2. Ethics Statement

All the procedures and the use of monkeys were approved (3 January 2011) by the Korea Research Institute of Bioscience and Biotechnology (KRIBB) Institutional Animal Care and Use Committee (Approval No. KRIBB-AEC-11010).

### 4.3. Total RNA Isolation and cDNA Preparation

Total RNA was extracted from five regions of the two matched-pair brain samples of control cynomolgus monkeys and those who had received intracerebroventricular injections of streptozotocin (icv-STZ) to obtain a total of 20 samples by using the RNeasy Mini kit (Qiagen, GmbH, Hilden, Germany), according to the manufacturer’s instructions. RNase-free DNase (Qiagen, GmbH, Hilden, Germany) was used to eradicate DNA contamination from the total RNA preparations. The RNA concentration and the absorbance ratio at 260 and 280 nm (A260/A280) were determined with a NanoDrop^®^ ND-1000UV-Vis Spectrophotometer (NanoDrop Technologies, Wilmington, DE, USA). The A260/A280 was 2.09–2.16 for all the samples (data not shown). To generate cDNA, 500 ng of total RNA was reverse-transcribed using the SuperScript III First-Strand Synthesis System (Invitrogen, Carlsbad, CA, USA) followed by RNase H treatment (Invitrogen, Carlsbad, CA, USA), according to the manufacturer’s instructions.

### 4.4. Primer Design and Standard Curve Analysis

Specific primer pairs for seven APP pathway-related genes, five tau phosphorylation-related genes using the Primer3 program (Table 2) [31]. Four reference genes were used for normalization of target genes from our previous study [19]. Gene sequences were obtained from data obtained in our previous large-scale transcriptome sequencing analysis of the cynomolgus monkey [32]. BLAST searches were performed to confirm the gene specificity of the primer sequences, and the results showed an absence of multi-locus matching at individual primer sites. Most primers spanned at least two exons or were designed to amplify exons separated by large introns, in order to avoid false-positive amplification of any contaminating genomic DNA in the RNA samples. The nucleotide sequences of the RT-PCR products for the 12 target genes and four reference genes were obtained using standard cloning and sequencing procedures (Figures S1 and S2). Briefly, RT-PCR products were separated on a 1.5% agarose gel, purified using the Expin™ Gel SV (GeneAll Biotechnology, Seoul, Korea), and cloned into the pGEM-T easy vector (Promega, Madison, WI, USA). Sequencing services were performed by Macrogen (Macrogen Inc., Seoul, Korea). Amplification efficiencies and correlation coefficients (*R*^2^ values) of the 16 genes were generated using the slopes of the standard curves obtained by performing RT-PCR using a 10-fold serial dilution series (Table 2). The amplification efficiency was calculated according to the formula: efficiency (%) = (10^(−1/slope)^ − 1) × 100, and the range for the real-time RT-PCR amplifications for all the tested genes was 81%–98%.

**Table 2:** Primers for the 12 genes of target genes and two reference genes and parameters derived from RT-qPCR data analyses.

Gene Symbol	Gene Name	Primer * Forward (F)/Reverse (R) 5'–3'	Exon(s)	Amplicon Size (bp)	PCR Efficiency (%)	*R*^2^	NTC ** (Cq)
*ADAM10*	ADAM metallopeptidase domain 10	F: TGCAAACTGAAACCTGGGAA	11	121	92	0.98922	31.3
		R: TTCCTTCCCTTGCACAGTCT	12				
*ADAM17*	ADAM metallopeptidase domain 17	F: CATGAAT/GGCAAATGTGAGAAAC	15/16	168	81	0.99394	33.8
		R: TGGACAAGAATGCTGAAAGGA	17				
*BACE1*	Beta-site APP-cleaving enzyme 1	F: CGGGTGGAGATCAATGGACA	5	194	82	0.99029	N.d.
		R: CACACCAGCTGCTCTCCTAG	7				
*PSEN2*	Presenilin 2 (Alzheimer disease 4)	F: CCGCTGCTACAAG/TTCATCC	5/6	143	93	0.99045	31.9
		R: TCCAGACAGTCAGCAAGAGG	7				
*NCSTN*	Nicastrin	F: CTGTGTTCGCCTGCTCAAC	2	123	92	0.99172	34.1
		R: GGGCCATCAGTCAATACCCA	3				
*APH1A*	Anterior pharynx defective 1 homolog A (C. elegans)	F: ACCTACTGACATCGGGACTG	5	140	96	0.99049	32.7
		R: GAGGCTGCGCTGAATACTTC	6				
*PSENEN*	Presenilin enhancer 2 homolog (C. elegans)	F: ACCTGTGCCGGAAGTACTAC	2	113	98	0.99198	36.9
		R: CTGTTCTGTGTAGGCTGGGA	3				
*DK5*	Cyclin-dependent kinase 5	F: CAGTGGCCCTCTATGACCAA	10	76	86	0.99153	N.d.
		R: CGTTCACCAGGGATGTTGTG	11				
*DK5R1*	Cyclin-dependent kinase 5, regulatory subunit 1 (p35)	F: GCTGCCTTGGAAGAGAATCG	2	94	91	0.99565	N.d.
		R: GTGCGTGATGTTGTTCTGGT	2				
*CAPN1*	Calpain 1, (mu/I) large subunit	F: ATGACCAGATCCAGGCCAAT	13	122	93	0.99090	N.d.
		R: CTCCTTCACGCTGATCTCCA	15				
*AKT1*	V-akt murine thymoma viral oncogene homolog 1	F: CCACGCTACTTCCTCCTCAA	3	159	92	0.99906	35.62
		R: GCGGATGATGAAGGTGTTGG	4				
*GSK3β*	Glycogen synthase kinase 3 beta	F: TCGCCATCAAGAAAGTATTGCA	2	94	86	0.99641	N.d.
		R: CGCAATCGGACTATGTTACAGT	3				
*ACTB*	Beta-actin	F: ACAGAGCCTCGCCTTTGC	1	160	92	0.99094	32
		R: CACGATGGAGGGGAAGAC	2				
*GAPDH*	Glyceraldehyde-3-phospate dehydrogenase	F: ACAACAGCCTCAAGATCGTCAG	6	112	90	0.99273	34.18
		R: ACTGTGGT/CATGAGTCCTTCC	7/8				
*RPS19*	Ribosomal protein S19	F: AGCTTGCTCCCTACGATGAG	3	174	93	0.99581	36.04
		R: GACGAGCCACACTCTTGGA	4				
*YWHAZ*	Tyrosine 3-monooxygenase/tryptophan 5-monooxygenase activation protein, zeta polypeptide	F: AGCAGATGGCTCGAGAATACA	2	185	97	0.99120	38.44
		R: GTCATCACCAGCGGCAAC	3				

* Where a primer spans two exons, the junctions are indicated by a virgule; ** No template control; N.d.: Not detected; Cq: quantification cycle.

### 4.5. RT-qPCR Amplification

RT-qPCR using SYBR Green was performed using a Rotor Gene Q thermocycler (Qiagen, GmbH, Hilden, Germany). For each reaction, 1 µL of cDNA was used as a template and added to 19 µL of reaction mixture containing 7 µL H_2_O, 10 µL Rotor Gene SYBR Green PCR mastermix (Qiagen, GmbH, Hilden, Germany), and 1 µL each of the forward and reverse primers (10 pmol). RT-qPCR amplification of the 16 genes was performed for 40 cycles of 94 °C for 5 s and 60 °C for 10 s. The amplification specificity of each RT-qPCR assay was confirmed by melting curve analysis. The temperature range for analysis of the melting curves was 55–99 °C for 5 s. As shown in Figure S3, each primer pair showed a single, sharp peak, thereby indicating that the primers amplified only 1 specific PCR product. No amplification from the no-template control (NTC) was observed for *BACE1*, *CDK5*, *CDK5R1*, *CAPN1*, and *GSK3*β genes, and although some was detected for other genes, amplification was only observed after 31 cycles (Table 2), and the minute amounts of primer dimers occurring did not affect the fluorescence level of the amplified target gene. Data were generated from three independent experiments. All the target genes were normalized for relative quantification by the normalization factor (NF) derived from geometric means delta-Cq (quantification cycles) of the ribosomal protein S (*RPS19*) and tyrosine 3-monooxygenase/tryptophan 5-monooxygenase activation protein ζ polypeptide (*YWHAZ*) of previous selected appropriate reference genes (Figure 2 and Figure 4) and control group and icv-STZ group were normalized by the NF of *RPS19* and *GAPDH* and NF of β-actin (*ACTB*) and glyceraldehyde-3-phosphate dehydrogenase (*GAPDH*), respectively (Figure 1 and Figure 3) [19]. All the experiments were performed in triplicate.

### 4.6. Statistical Analysis

The statistical significance of the difference was determined using the REST 2009 program, wherein the null hypothesis was tested by a Pair Wise Fixed Reallocation Randomization Test© with 2000 permutations (* *p* < 0.05 and ** *p* < 0.001) (Figure 2 and Figure 4) [33].

### 4.7. Minimum Information for Publication of Quantitative Real-Time PCR Experiments (MIQE) Guidelines

All the experiments were performed according to the Minimum Information for Publication of Quantitative Real-Time PCR Experiments (MIQE) guidelines [34].

## 5. Conclusions

In this study, quantitative analysis of seven APP pathway-related genes and five tau phosphorylation-related genes was performed on tissues from five brain regions obtained from control and icv STZ-treated cynomolgus monkeys. Our results indicate that the genes’ expression levels could be affected by injection of STZ. Almost all genes showed significantly increased expression levels in the precuneus and occipital cortex in icv STZ-treated cynomolgus monkeys. These expression changes may lead to increases in risk factors for sAD, including accumulation of Aβ and hyperphosphorylated tau. Finally, to further verify that this monkey model is appropriate for the study of sAD, more functional investigations of various AD-related genes in the icv STZ-treated monkey are required.

## Supplementary Materials

Supplementary materials can be found at http://www.mdpi.com/1422-0067/16/02/2386/s1.
